# Biological interactions and cooperative management of multiple species

**DOI:** 10.1371/journal.pone.0180189

**Published:** 2017-06-29

**Authors:** Jinwei Jiang, Yong Min, Jie Chang, Ying Ge

**Affiliations:** 1 College of Computer Science and Technology, Zhejiang University of Technology, Hangzhou, Zhejiang, China; 2 College of Life Sciences, Zhejiang University, Hangzhou, Zhejiang, China; 3 Institute of Environment Sciences, Department of Biology Science, University of Quebec at Montreal, Montreal, Quebec, Canada; Ecole Polytechnical university, CHINA

## Abstract

Coordinated decision making and actions have become the primary solution for the overexploitation of interacting resources within ecosystems. However, the success of coordinated management is highly sensitive to biological, economic, and social conditions. Here, using a game theoretic framework and a 2-species model that considers various biological relationships (competition, predation, and mutualism), we compute cooperative (or joint) and non-cooperative (or separate) management equilibrium outcomes of the model and investigate the effects of the type and strength of the relationships. We find that cooperation does not always show superiority to non-cooperation in all biological interactions: (1) if and only if resources are involved in high-intensity predation relationships, cooperation can achieve a win-win scenario for ecosystem services and resource diversity; (2) for competitive resources, cooperation realizes higher ecosystem services by sacrificing resource diversity; and (3) for mutual resources, cooperation has no obvious advantage for either ecosystem services or resource evenness but can slightly improve resource abundance. Furthermore, by using a fishery model of the North California Current Marine Ecosystem with 63 species and seven fleets, we demonstrate that the theoretical results can be reproduced in real ecosystems. Therefore, effective ecosystem management should consider the interconnection between stakeholders’ social relationship and resources’ biological relationships.

## Introduction

Healthy ecosystems usually contain a variety of renewable natural resources, and these resources interact through diverse biological processes [1]. If focusing on a single resource or treating whole ecosystems as inseparable units, ecosystem management is often modeled as a typical public goods game [2–4], and the natural attributes of an ecosystem are often overlooked or proven to be secondary if they do not take into account technical factors [5–7]. However, for the management of interacting resources, this hypothesis is questionable [8].

The recent emergence of ecosystem-based management emphasizes the contribution of biological interactions in the sustainable use of the multiple resources embedded in an ecosystem [9]. Theoretical studies on the economics of interdependent resources began in the 1970s and predominantly compared the free access equilibrium and the social optimum in ecosystems [10–13]. However, none of these papers analyzed strategic conflicts and interactions [3,4]. Since 1979, game theory has become the major tool for analyzing the cooperative (joint) versus non-cooperative (separate) management of ecologically interdependent resources [8,14–17]. Nevertheless, almost all of these works have been based on predation relationships or at least have not effectively distinguished between different types of biological interactions.

The biological interactions in an ecosystem are various and can be classified by the mechanism of the interaction or the features of their effects [18]. If further considering indirect effects, the interactions of the ecosystem services involved in ecosystem management will be more complex [1,19]. Therefore, the study of predation relationships does not adequately reflect such complex relationships between resources and ecosystem services. Currently, the response of cooperative management to different biological interactions is still unclear.

## Materials and methods

### Two-species bioeconomic model

Here, we consider a theoretical 2-species biological model, where the two species exhibit logistic growth [20] and the interaction between the species is described by the type II functional response [8]:

$$\begin{array}{c} \frac{\text{d}B_{1}}{\text{d}t}=G_{1}B_{1}(1-\frac{B_{1}}{K_{1}})+\frac{\beta_{21}B_{1}B_{2}}{1+B_{2}}-h_{1}B_{1}\\ \frac{\text{d}B_{1}}{\text{d}t}=G_{2}B_{2}(1-\frac{B_{2}}{K_{2}})+\frac{\beta_{12}B_{1}B_{2}}{1+B_{1}}-h_{2}B_{2} \end{array}$$ (1)

where *B*_*i*_ is the biomass of species *i*. The parameter values have the following meaning: *G*_*i*_ is the mass-specific maximum growth rate of species *i*, *K*_*i*_ is the carrying capacity for species *i*, and *β*_*ij*_ is the interaction strength of *i* affecting *j*. *β*_*ij*_ > 0 means species *i* benefits species *j*, *β*_*ij*_ < 0 means species *i* is harmful to species *j*, and if *β*_*ij*_ = 0, species *i* has no effect on species *j*. Management actions or harvests are assumed to directly affect to the harvest mortality of species *i* (*h*_*i*_), and the combination of two harvest rates forms management strategy **H**. In the simulating analysis, we set *G*_1_ = 1, *G*_2_ = 0.8, and *K*_1_ = *K*_2_ = 1.

We chose this simple model for three reasons. First, the combination of *β*_12_ and *β*_21_ can define different types of biological interactions in the ecosystem, including competition (*β*_12_ < 0 and *β*_21_ < 0), mutualism (*β*_12_ > 0 and *β*_21_ > 0), and predation (*β*_12_ > 0 and *β*_21_ < 0, and vice versa). The continuous variation of *β*_*ij*_ can also simulate the change in interaction strength. Second, exact solutions of steady states (B*) could be analytically derived for the model. Consequently, it was feasible to quantify management outcomes in a reasonable amount of computing time. Third, the type II functional response is sufficiently generally applicable in describing various interactions within ecosystems [8,21,22].

Suppose that there are two agents (i.e., owners), each of whom gains ecosystem services by catching only their own species. The ecosystem service provided by the system or private payoff of an agent is defined as harvest earnings after removing prime costs. Harvest earning is described as the fixed market price multiplying the harvest yield, and the cost is defined by stock effects [23]:

$$\begin{array}{c} p_{i}(B_{i},h_{i})=\text{revenue}-\text{cost}\\ =q_{i}E_{i}-\frac{a_{i}}{B_{i}}E_{i} \end{array}$$ (2)

where *q*_1_ = *q*_2_ = 1 is the constant market price per unit biomass, *E*_*i*_ = *B*_*i*_*h*_*i*_ is the harvested quantity, and the coefficient *a*_*i*_ = 0.1*S*_*i*_*q*_*i*_, which will cause individual marginal profits drop to zero when the resource abundance goes down to 10% of its steady state (*S*_*i*_) without agent extractions [8].

### North California Current Marine Ecosystem model

The North California Current Marine Ecosystem (NCCME) is an eastern boundary current upwelling zone on the western coast of the USA [24,25] that has 63 living functional groups and 7 fishing fleets (i.e., agents): bottom trawl; shrimp trawl; hake trawl; line, pot and trap; salmon fishery; crab pot; and other small fisheries. All but the crab pot fleet target multiple species. The dynamics of the NCCME were simulated by EcoSim (S1 Appendix), which has been used widely for modeling marine food webs and for addressing questions about ecosystem management [26,27]. The management actions of fleets were characterized by the level of annual fishing effort (**H**). We measured each fleet’s ecosystem services or private payoff (*p*_*i*_) as its steady-state annual revenue (equal to species-specific equilibrium annual yields times their market prices), subtracting the cost related to the base effort (hence, if the effort is increased, the variable costs are assumed to increase proportionally). The group information, diet composition, fleet information and off-vessel prices of the NCCME can be found in S1–S5 Tables. The steady-state (B*) of the NCCME based on given fishing efforts can be evaluated by simulating long-term system dynamics (> 100 years).

To reflect different interaction types between the species targeted by the fleets, we consider three scenarios based on two carefully selected fleets and keep other fleets at the base effort (equal to 1 in EcoSim). For the scenario of competing resources, we consider the hake trawl and salmon fishery because the diets of hake and salmon are very similar (S2 Table). For the scenario of a predation relationship, we consider bottom trawl and hake trawl. Most of the species targeted by the bottom trawl (e.g., arrowtooth) are fed upon by hake (S2 Table), which is the major target of the hake trawl. Because most of the targeted species are similar between the bottom trawl and the line, pot and trap (S3 and S4 Tables), the two fleets can be considered in the scenario of exploiting mutual resources.

### Game theoretic analysis framework

**Fig 1:**
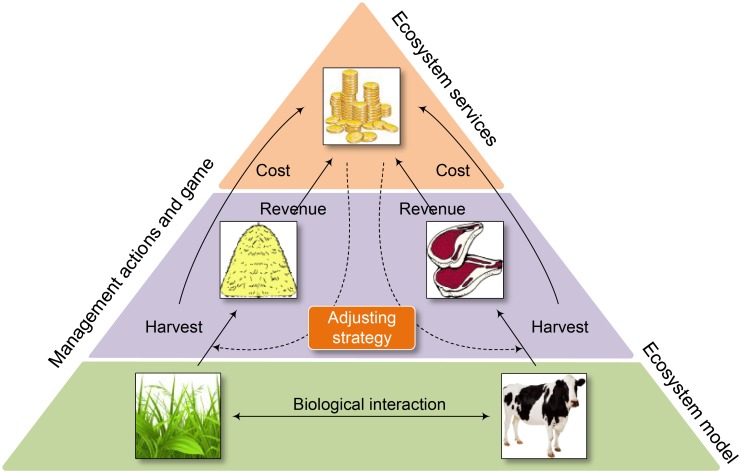
The demonstration of the game theoretic framework. The framework is based on an ecosystem dynamic model simulating changes in resources according to the agents' management actions and biological interactions. The management action (i.e., harvest) generates revenue for each agent and results in the related costs. Based on the management regimes (cooperation or non-cooperation), agents can adjust their actions according to joint social or private benefits.

Based on the above ecosystem models, we can construct a classical game theoretic framework (Fig 1) to compare cooperative (or joint) and non-cooperative (or separate) management equilibrium outcomes of the model and investigate the effects of the type and strength of the relationships.

Under non-cooperative management, the goal of each agent is to maximize its long-term private payoff from harvest (i.e., at steady state B*):

$$obj_{i}=\underset{h_{i}}{\text{max}}p_{i}(B_{i}^{*}(H),h_{i})$$ (3)

where **H** is the set of strategies (i.e., harvest rates). The above targets will constitute a typical non-cooperative strategic game. A reasonable equilibrium for the game is the Nash equilibrium strategy (**H**_N_), at which no agent can gain more payoff by changing its actions [8].

For cooperative management, the social objective is to maximize the sum of all agents’ private payoffs (i.e., joint social value):

$$obj_{\text{joint}}=\underset{H}{\text{MAX}}\sum_{i=1}^{n}p_{i}(B_{i}^{*}(H),h_{i})$$ (4)

Attributed to this goal, cooperation will draw out an optimal strategy (**H**_C_) for long-term joint social value.

To identify the Nash equilibrium and the optimal strategy, a direct search algorithm and the fixed-point iteration are performed to identify the combination of harvest rates for each agent. Direct search is a family of numerical optimization methods that does not require the gradient of the objective function. Here, the generalized pattern search algorithm [28] is adopted to identify equilibrium. The algorithm starts with an initial point (i.e. solution) and searches for a set of points (called a mesh) around the current solution. The mesh is formed by adding the current point to a scalar multiple of a set of vectors called a “pattern” (generalized pattern search algorithm uses the uses fixed direction vectors). If the pattern search algorithm finds a point in the mesh that improves the objective function at the current point, the new point becomes the current point, and the algorithm is repeated until meeting the terminal condition of the algorithm. The objective function for the Nash equilibrium is

$$obj_{\text{Nash}}=\underset{H}{\text{MIN}}\sum_{i=1}^{n}\left(p_{i}(H)-\underset{h_{i}}{\text{MAX}}p_{i}(H^{\prime })\right)$$ (5)

where **H**′(*j*) = **H**(*j*) for *j* ≠ *i*, and **H**′(*j*) = *h*_*i*_ for *j* = *i*. In this way, no agent can gain more by solely changing its own harvest rate. In addition, the objective function for cooperative game is in Eq 4. For each objective, the algorithm is performed using multiple initial solutions and other conditions (e.g. the function or mesh tolerance of direct search) to verify convergence.

Under the fixed-point iteration approach, the harvest rates of all agents but one are fixed, with the remaining agent choosing the management action that maximizes the private payoff or joint social value. The process is replicated for each agent and then repeated in its entirety until there are no changes in harvest rates (≤ 10^−4^).

By taking the Nash strategy as a baseline, we can calculate the normalized joint social value (JSV), total resource abundance (TRA), and resource evenness (EVE) of cooperation as the percentage of the change in the value under joint versus separate management:

$$\begin{array}{c} \text{JSV}=\frac{\sum p_{i}(H_{\text{N}})-\sum p_{i}(H_{\text{C}})}{\sum p_{i}(H_{\text{C}})}\times 100\%\\ \text{TRA}=\frac{\sum B_{i}^{*}(H_{\text{N}})-\sum B_{i}^{*}(H_{\text{C}})}{\sum B_{i}^{*}(H_{\text{C}})}\times 100\%\\ \text{EVE}=\frac{E(H_{\text{C}})-E(H_{\text{N}})}{E(H_{\text{N}})}\times 100\% \end{array}$$ (6)

where *E* is the Shannon index of species evenness:

E(X)=∑i=1nηiln(ηi)ln(1n), and ηi=Bi*(H)∑Bj*(H) (7)

where *η*_*i*_ is the proportion of equilibrium biomass of species *i* to the total biomass when applying the set of strategies **H**. We will use these three indictors to evaluate the effect of interaction types and strength.

## Results

**Fig 2:**
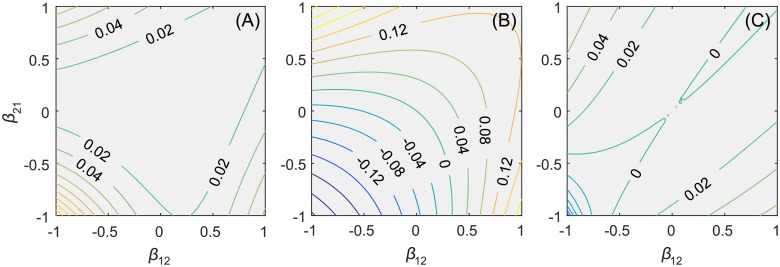
The effects of biological interactions in the two-species bioeconomic model. Contour plot of the responses of normalized (A) joint social value, (B) total resource abundance, and (C) diversity to the interaction type and strength between two species. *β*_*ij*_ is the interaction direction and strength of species *i* affecting species *j*. *β*_*ij*_ > 0 indicates that species *i* benefits species *j*, while *β*_*ij*_ < 0 indicates that species *i* is harmful to species *j*. The positive normalized ratio indicates that cooperative management is superior to non-cooperative management in this indicator and related interacting styles and vice versa.

The goal of joint management is to maximize the joint social value; thus JSV is consistently positive in all interspecific relationships and can be up to 20% (Fig 2A) in the two-species model. In approximately 60% of weak relationships and almost all mutual relationships, there is no obvious economic advantage of joint management (JSV is less than 2%). Only when the two resources show high competition and predation (*β*_12_ and *β*_21_ have at least one negative value, and both absolute values are greater than approximately 0.5), cooperation has an obvious economic advantage (> 5%), and the stronger the relationship, the more obvious the advantages. For competitive relationships, the JSV can reach 20%, while for predation relationships, it can reach 10%. This result suggests that the economic advantage of cooperation is mainly related to the negative interactions between resources.

**Fig 3:**
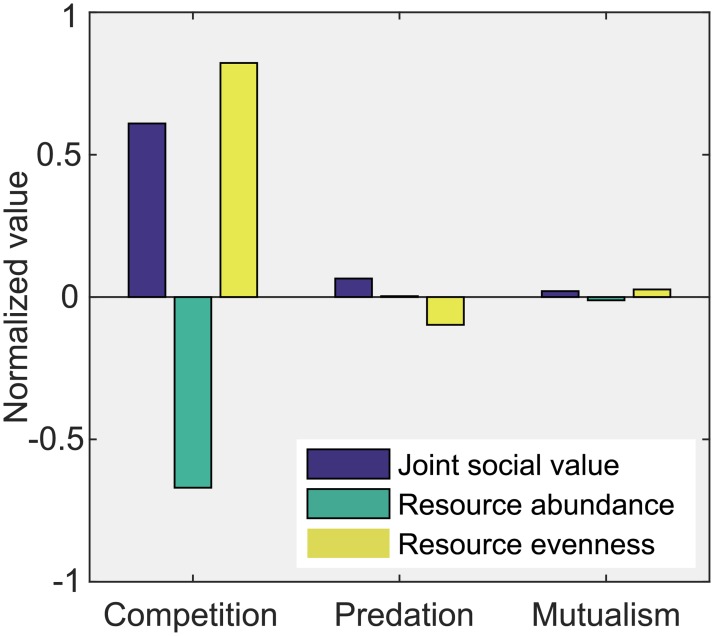
The effects of biological interactions in the North California Current Marine Ecosystem (NCCME) model. The normalized outcomes of cooperative management, quantified as the percentage change in joint social value, resource abundance, and evenness under cooperative versus non-cooperative management in three scenarios (competition, predation, and mutualism) based on the NCCME.

The NCCME model produces similar patterns of joint social value (Fig 3). For the competition scenario, the JSV from the hake trawl and salmon fishery is positive and reaches 60.96%. The JSVs of the predation and mutualism scenarios are 6.50% and 2.07%, respectively. The JSVs of three scenarios also increase with the intensity of negative interactions between resources.

In contrast to the consistent positive effects on the joint social value, cooperation has distinct impacts on resource abundance in various biological interactions (Fig 2B). In approximately 75% of relationships, the difference in resource abundance due to joint or separate management is greater than 5%. Moreover, cooperation led to both increases and decreases in resource abundance. The TRA in competitive and weak predation relationships leads to the resource abundance being less in joint management than that in separate management, with a minimum of -30%. In contrast, the TRA in strong predation relationships can significantly enhance resource richness (> 10%) up to 30%. For mutualistic relationships, although joint management has no significant advantage in terms of joint social value, it can still effectively enhance resource abundance. When *β*_12_ and *β*_21_ tend toward 1.0, the TRA can exceed 12%. Therefore, “asymmetric” interactions between resources, e.g., predation relationships, are the key to enhancing resource abundance through joint management.

In the NCCME model, joint management generates a relatively high loss in resource abundance (TRA = -66.98%) of related species under the competition scenario (Fig 3). The TRA values for the predation and mutualism scenarios are negligible, 0.03% and -1.15%, respectively (Fig 3). This tendency is consistent with the results of the theoretical analysis, except for the negative TRA in the mutualism scenario.

Compared with the previous two indicators, cooperation has relatively minor impacts on resource evenness across all interactions (Fig 2C). Joint management can increase evenness with predation relationships (EVE up to 8%). Conversely, for a small number of strong competitive relationships (approximately 1%), cooperation had significant negative effects (EVE < -3%) on evenness, with the lowest value being -14%. For mutual resources, cooperation will result in a slight decline in evenness (EVE < -1%). Furthermore, the impact of joint management on evenness is consistent with the effect on joint social value and abundance, which are obviously affected by the intensity of biological interactions.

The scenarios of the NCCME model display a different pattern of EVE (Fig 3). In the competition scenario, joint management leads to a high increase in resource evenness (EVE = 82.21%). Conversely, EVE in the predation scenario is negative (EVE = -9.76%). Moreover, joint management has a minor effect on mutual resources (EVE = 2.66%). The scenario results are completely different from those of the conclusion of the theoretical model.

## Discussion

In ecological economics, there has been a long history of concern about the management of interacting resources [8,10–12,14–16]. In this paper, we provide higher resolution to the effects of biological interactions on cooperative management. The above results demonstrate that the intensity and type of interspecific relationships among multiple resources play a critical role in ecosystem management. First, the difference between cooperative and non-cooperative management is positively correlated with interaction strength. In fact, when the biological interaction is weak, the resources are relatively independent. Thus, cooperation among multiple agents is meaningless. Second, the type of biological interaction between resources determines whether cooperation can improve the JSV while also leading to a higher TRA and DIV. Joint management can only achieve win-win results in predation relationships; however, for competitive relationships, it can significantly enhance the JSV but at the expense of the TRA and DIV. Third, mutual resources seem to not require cooperation because the sustainability of one can be transmitted to the other through mutual relationships. However, the model analysis shows that cooperation was able to improve the TRA to a higher level while maintaining a similar JSV. In sum, the measurement of the interactions between resources or ecosystem services should be a prerequisite for joint management.

**Fig 4:**
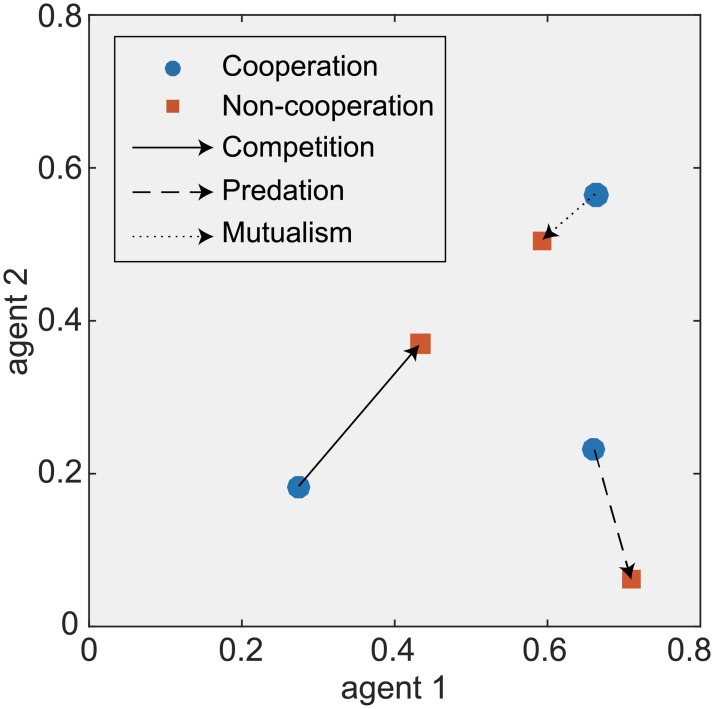
The difference in agents’ strategies between cooperative and non-cooperative management based on various interactions. The figure uses arrows to connect optimal strategies (blue cycles) and Nash equilibrium strategies (orange squares) in three scenarios (competition, predation and mutualism) respectively.

By comparing Nash (**H**_N_) and optimal (**H**_C_) strategies, we can explore how biological interactions affect agents’ decision making under different management regimes (Fig 4). For competitive resources, an agent increasing its harvest rate will reduce the abundance of the corresponding resource, which benefits the other competing resource. As a result, agents tend to adopt relatively lower harvest rates for the sake of self-interest. However, cooperation concurrently increases the harvesting rate of both resources to improve the JSV, whereas it reduces TRA and DIV. For predation relationships, cooperation reduces the harvest rate of the prey and increases the harvest rate of the predator. This change can increase the abundance of predators, thus contributing to the conservation of overall abundance and diversity. For the mutual relationship, the difference between the two strategies is slight, and cooperation moderately reduces the harvest rates of both resources. Nonetheless, by increasing the abundance of resources, the harvested resources are increased, thus ensuring the provisioning of ecosystem services. The results indicate that there is a complex coupling between resources’ biological interactions and agents’ strategic interactions, which will significantly affect the performance of management regimes.

By comparing results from the theoretical two-species and empirical NCCME models, we find they are not exactly consistent, especially in terms of resource biomass and diversity. This inconsistency can be explained based on three aspects. First, the target of the game is only the market value or provisioning services for stakeholders and does not consider resource abundance, non-market value or other services provided by ecosystems. As a result, the results regarding the long-term joint social value of cooperative management is highly consistent in the theoretical and empirical models. Second, in the theoretical model, the relationship between agents and species is a one-to-one correspondence; thus, the interspecific relationships can exactly reflect the interactions in the ecosystem services gained by agents. In contrast, in the NCCME model, all agents but the crab pot fishery target multiple species; thus, the relationships between the major species harvested cannot completely reflect the complex interactions between the ecosystem services provided to agents. Third, the multiple species targeted by an agent also display complex relationships; consequently, a change in an agent’s fishing effort could lead to a complex change in the diversity of related species. In fact, the direct factor affecting decision making is the relationships between ecosystem services obtained by agents rather than interspecific relationships. In the theoretical model, the two relationships are straight; however, there are complicated in real-world cases. Recently, researchers have developed many tools to evaluate the interactions between multiple ecosystem services [1], which has provided a good foundation for verifying and applying our results in real-world ecosystems.

For multispecies management, our work reveals a complete chain for realizing sustainable exploitation. The chain involves three types of interactions, including resource (interspecific) interactions, ecosystem services interactions, and strategic interactions. Resource interactions determine the interactions among ecosystem services, which further affect strategic interactions. The chain emphasizes the effect of resource interactions on the performance of management regimes. To seek cost-efficient management strategies for the multispecies ecosystem, we need to take full account of the chain to evaluate and compare different management regimes for various resource systems. For this chain, our analysis of a theoretical two-species model provides a general connection between interspecific relationships and cooperative management. Moreover, the analysis of the empirical NCCME model supports the general connection and shows the potential complexity of the chain.

## Conclusion

Our results show that the economic advantage of cooperation is predominantly determined by the intensity of negative interactions between resources or ecosystem services, but the ecological advantages (abundance and diversity) are predominantly attributed to the asymmetrical interactions (e.g., predation and parasitism). Therefore, the theoretical analysis provides a potential method for predicting the value and feasibility of cooperative management based on the measurement of biological interactions.

## Supporting information

S1 Appendix The EcoSim model of the North California Current Marine Ecosystem (NCCME). (DOCX)

S1 Table Group information for the NCCME. (DOCX)

S2 Table Diet composition in the NCCME. (DOCX)

S3 Table Fleet landing in the NCCME. (DOCX)

S4 Table Fleet discarding in the NCCME. (DOCX)

S5 Table Market price (US dollars). (DOCX)
