# Supplementary material for: Biological interactions and cooperative management of multiple species
Source: PLoS One. 2017 Jun 29;12(6):e0180189. doi: 10.1371/journal.pone.0180189 (PMC5491148; doi:10.1371/journal.pone.0180189)
Supplement: S1 Appendix — (DOCX) [file pone.0180189.s001.docx]

**Appendix 1. The Ecosim model of North California current marine ecosystem (NCCME)**

1.1 Ecosim basic

The Ecosim dynamical differential equations are derived from the Ecopath master equation (Pauly et al. 2000):

$\frac{dB_{i}}{dt}=g_{i}\sum_{j} Q_{ji}-\sum_{j} Q_{ij}+I_{i}-(MO_{i}+F_{i}+e_{i})B_{i}$ (eqn1)

where *g_i_* is the production/consumption ratio, *MO_i_* the non-predation natural mortality rate, *F_i_* is fishing mortality rate, *e_i_* is emigration rate, *I_i_* is immigration rate. The consumption rates, *Q_ji_*, are calculated based on the ‘foraging arena’ concept (Walters et al. 1997), where *B_i_*’s are divided into vulnerable and invulnerable components, and it is the transfer rate (*v_ij_*) between these two components that determines if control is top-down (i.e., Lotka-Volterra), bottom-up (i.e., donor-driven), or of an intermediate type.

1.2 Basic information of NCCME

All these data and related Ecosim model are published in (Field 2004) and available in the website of EcoBase: <http://sirs.agrocampus-ouest.fr/EcoBase/>. We list important data in the S1-5 Table.

**LITERATURE CITED**

Field, J. 2004. Application of ecosystem-based fishery management approaches in the Northern California Current.

Pauly, D., V. Christensen, and C. Walters. 2000. Ecopath, Ecosim, and Ecospace as tools for evaluating ecosystem impact of fisheries. *ICES Journal of Marine Science* 57(3):697–706.

Walters, C., V. Christensen, and D. Pauly. 1997. Structuring dynamic models of exploited ecosystems from trophic mass-balance assessments. *Reviews in Fish Biology and Fisheries* 7(2):139–172.
