# Supplementary material for: Biological interactions and cooperative management of multiple species
Source: PLoS One. 2017 Jun 29;12(6):e0180189. doi: 10.1371/journal.pone.0180189 (PMC5491148; doi:10.1371/journal.pone.0180189)
Supplement: S1 Table — (DOCX) [file pone.0180189.s002.docx]

**Table 1. Group information of NCCME**

|  | Group name | Trophic level | Biomass in habitat area (t/km^2^) | Biomass (t/km^2^) | Production / biomass (computed) (/year) | Consumption / biomass (/year) | Ecotrophic Efficiency | Production / consumption | Biom. accumul. (t/km^2^/year) | Biom. acc. rate (/year) |
| --- | --- | --- | --- | --- | --- | --- | --- | --- | --- | --- |
| 1 | phytoplankton | 1 | 55.15 | 55.15 | 120 |  | 0.400934 |  |  |  |
| 2 | infauna | 2 | 35.7 | 35.7 | 2.5 | 12 | 0.840095 | 0.208333 |  |  |
| 3 | amphipods | 2 | 4.276248 | 4.27625 | 3.5 | 22 | 0.8 | 0.159091 |  |  |
| 4 | epibenthic | 2.45 | 12.09117 | 12.0912 | 2 | 10 | 0.8 | 0.2 |  |  |
| 5 | micro-zoop | 2 | 3.693789 | 3.69379 | 100 | 300 | 0.8 | 0.333333 |  |  |
| 6 | copepods | 2.2 | 15.61595 | 15.6160 | 14 | 70 | 0.8 | 0.2 |  |  |
| 7 | euphausiids | 2.11 | 25.24237 | 25.2424 | 8 | 40 | 0.8 | 0.2 |  |  |
| 8 | carniv-zoops | 3.144228 | 7.137304 | 7.13730 | 2 | 10 | 0.8 | 0.2 |  |  |
| 9 | small jellies | 2.25 | 1.11369 | 1.11369 | 9 | 30 | 0.8 | 0.3 |  |  |
| 10 | large jellies | 3.161385 | 1.034882 | 1.03488 | 3 | 12 | 0.8 | 0.25 |  |  |
| 11 | pandalid shp | 2.748827 | 1.50067 | 1.50067 | 2 | 10 | 0.8 | 0.2 |  |  |
| 12 | benthic shp | 3.041056 | 1.54762 | 1.54762 | 2.5 | 12 | 0.8 | 0.208333 |  |  |
| 13 | dungeness | 3.455873 | 1.0275 | 1.0275 | 0.75 | 3.8 | 0.641679 | 0.197368 |  |  |
| 14 | tanner crb | 2.9631 | 0.761439 | 0.76144 | 0.3 | 1.5 | 0.8 | 0.2 |  |  |
| 15 | cephalopods | 3.616686 | 1.954489 | 1.95449 | 2 | 6 | 0.8 | 0.333333 |  |  |
| 16 | forage fish | 3.173189 | 25.71446 | 25.7145 | 1.5 | 6 | 0.8 | 0.25 |  |  |
| 17 | mesopelagics | 3.239981 | 6.550208 | 6.55021 | 0.6 | 3 | 0.8 | 0.2 |  |  |
| 18 | benthic fish | 3.276965 | 3.706484 | 3.70648 | 0.5 | 2.5 | 0.8 | 0.2 |  |  |
| 19 | macrourids | 3.65601 | 0.468 | 0.468 | 0.2 | 1 | 0.314275 | 0.2 |  |  |
| 20 | sardine | 2.833 | 1 | 1 | 0.5 | 5 | 0.931489 | 0.1 | 0.2 | 0.2 |
| 21 | mackerel | 3.478515 | 1.78 | 1.78 | 0.35 | 6 | 0.148464 | 0.058333 |  |  |
| 22 | salmon | 4.063568 | 0.418 | 0.418 | 0.932492 | 5.818928 | 0.728486 | 0.160252 |  |  |
| 23 | hake | 3.570522 | 28.925 | 28.925 | 0.18 | 1.956522 | 0.690628 | 0.092 | -2.9 | -0.1002 |
| 24 | skates | 4.022979 | 0.421 | 0.421 | 0.2 | 2 | 0.782629 | 0.1 |  |  |
| 25 | dogfish | 4.052045 | 1 | 1 | 0.2 | 2.5 | 0.387725 | 0.08 |  |  |
| 26 | sablefish | 4.065117 | 1.472 | 1.472 | 0.086 | 2.1 | 0.89579 | 0.040952 | -0.03994 | -0.0271 |
| 27 | juv rock | 3.266712 | 0.616277 | 0.61628 | 1.5 | 6 | 0.8 | 0.25 |  |  |
| 28 | POP | 3.338012 | 0.298 | 0.298 | 0.082 | 2.07 | 0.723714 | 0.039614 | -0.014 | -0.0469 |
| 29 | canary | 3.181332 | 0.2143 | 0.2143 | 0.113 | 1.66 | 0.777113 | 0.068072 | -0.02593 | -0.1209 |
| 30 | widow | 3.523815 | 1.486 | 1.486 | 0.163 | 2.2 | 0.43283 | 0.074091 | -0.117 | -0.0787 |
| 31 | yellowtail | 3.553986 | 1.43254 | 1.43254 | 0.146 | 1.7 | 0.810232 | 0.085882 | 0.004563 | 0.00319 |
| 32 | black | 3.976977 | 0.2399 | 0.2399 | 0.129 | 2.01 | 0.553955 | 0.064179 | -0.018 | -0.0750 |
| 33 | shelf rock | 3.737982 | 0.828 | 0.828 | 0.125 | 2.2 | 0.656239 | 0.056818 | -0.041 | -0.0495 |
| 34 | slope rock | 3.288872 | 0.585 | 0.585 | 0.06 | 1.91 | 0.860156 | 0.031414 | -0.032 | -0.0547 |
| 35 | ssthorny | 3.918083 | 0.337 | 0.337 | 0.08 | 0.47 | 0.834981 | 0.170213 | -0.023 | -0.0682 |
| 36 | lsthorny | 3.731195 | 1.72 | 1.72 | 0.055 | 0.35 | 0.886625 | 0.157143 |  |  |
| 37 | juv thorny | 3.369622 | 0.413927 | 0.41393 | 0.5 | 2.5 | 0.8 | 0.2 |  |  |
| 38 | juv round | 3.232603 | 0.234461 | 0.23446 | 1.5 | 5.125006 | 0.8 | 0.292683 |  |  |
| 39 | lingcod | 4.32794 | 0.522 | 0.522 | 0.3 | 2.4 | 0.165515 | 0.125 | -0.02 | -0.0383 |
| 40 | juv flat | 3.105616 | 1.153836 | 1.15384 | 1 | 4 | 0.8 | 0.25 |  |  |
| 41 | english | 3.182485 | 0.58 | 0.58 | 0.35 | 2.12 | 0.895245 | 0.165094 | 0.011 | 0.01897 |
| 42 | petrale | 4.077654 | 0.326 | 0.326 | 0.36 | 1.7 | 0.516461 | 0.211765 |  |  |
| 43 | small flat | 3.390298 | 3.886654 | 3.88665 | 0.5 | 2.5 | 0.8 | 0.2 |  |  |
| 44 | rex | 3.083022 | 0.4 | 0.4 | 0.5 | 2.12 | 0.81618 | 0.235849 | 0.006 | 0.015 |
| 45 | dover | 3.062899 | 1.394 | 1.394 | 0.12 | 1.07 | 0.590198 | 0.11215 | -0.072 | -0.0517 |
| 46 | arrowtooth | 4.325609 | 0.325 | 0.325 | 0.335 | 2.12 | 0.824175 | 0.158019 |  |  |
| 47 | halibut | 4.340346 | 0.156 | 0.156 | 0.335 | 2.12 | 0.477424 | 0.158019 | 0.006 | 0.03846 |
| 48 | albacore | 4.306535 | 0.014 | 0.014 | 0.36 | 7.3 | 0.637173 | 0.049315 |  |  |
| 49 | coastal sharks | 4.379432 | 0.05 | 0.05 | 0.18 | 2.8 | 0.490172 | 0.064286 |  |  |
| 50 | shearwaters | 4.19399 | 0.0029 | 0.0029 | 0.1 | 138 | 0 | 0.000725 |  |  |
| 51 | murres | 4.185557 | 0.0087 | 0.0087 | 0.1 | 129 | 0.28046 | 0.000775 |  |  |
| 52 | gulls | 4.067889 | 0.002 | 0.002 | 0.12 | 122 | 0 | 0.000984 |  |  |
| 53 | orcas | 4.877371 | 0.0002 | 0.0002 | 0.02 | 11.15 | 0 | 0.001794 |  |  |
| 54 | toothed whales | 4.383743 | 0.052 | 0.052 | 0.07 | 28.85 | 0.085769 | 0.002426 |  |  |
| 55 | sperm whales | 4.640937 | 0.037 | 0.037 | 0.02 | 6.61 | 0.189378 | 0.003026 | 0.0001 | 0.00270 |
| 56 | harbor seals | 4.415618 | 0.014 | 0.014 | 0.084 | 17.44 | 0.189031 | 0.004817 | 0.0002 | 0.01428 |
| 57 | sea lions | 4.477036 | 0.0377 | 0.0377 | 0.074 | 16.38 | 0.219191 | 0.004518 | 0.0005 | 0.01326 |
| 58 | fur seals | 4.467831 | 0.0047 | 0.0047 | 0.091 | 39.03 | 0.312836 | 0.002332 |  |  |
| 59 | grey whales | 3.01125 | 0.033 | 0.033 | 0.037 | 8.87 | 0.136691 | 0.004171 | 0.0001 | 0.00303 |
| 60 | baleen whales | 3.616275 | 0.16 | 0.16 | 0.037 | 7.58 | 0.22348 | 0.004881 | 0.0011 | 0.00688 |
| 61 | climateforcer | 1 | 1 | 1 | 1 |  | 0 |  |  |  |
| 62 | oceanproduction | 1 | 1 | 1 | 1 |  | 0 |  |  |  |
| 63 | fishery offal | 1 | 5 | 5 |  |  | 0.141409 |  |  |  |
| 64 | pelagic detritu | 1 | 10 | 10 |  |  | 0.148713 |  |  |  |
| 65 | Detritus | 1 | 10 | 10 |  |  | 0.186273 |  |  |  |
