# Supplementary material for: Biological interactions and cooperative management of multiple species
Source: PLoS One. 2017 Jun 29;12(6):e0180189. doi: 10.1371/journal.pone.0180189 (PMC5491148; doi:10.1371/journal.pone.0180189)
Supplement: S2 Table — (DOCX) [file pone.0180189.s003.docx]

**Table 2. Diet composition of NCCME**

| Pred^*^ | Prey | | Diet | | Pred^*^ | | Prey | | | Diet | Pred^*^ | | Prey | | | Diet | Pred^*^ | | | Prey | | Diet | Pred^*^ | | | Prey | | Diet |
| --- | --- | --- | --- | --- | --- | --- | --- | --- | --- | --- | --- | --- | --- | --- | --- | --- | --- | --- | --- | --- | --- | --- | --- | --- | --- | --- | --- | --- |
| 2 | 3 | | 0.43 | | 2 | | 10 | | | 0.25 | 2 | | 11 | | | 0.4 | 2 | | | 12 | | 0.4 | 2 | | | 13 | | 0.792 |
| 2 | 17 | | 0.3 | | 2 | | 18 | | | 0.15 | 2 | | 23 | | | 0.02 | 2 | | | 25 | | 0.02 | 2 | | | 26 | | 0.009 |
| 2 | 34 | | 0.05 | | 2 | | 35 | | | 0.16 | 2 | | 39 | | | 0.575 | 2 | | | 40 | | 0.34 | 2 | | | 42 | | 0.15 |
| 2 | 43 | | 0.55 | | 2 | | 44 | | | 0.85 | 2 | | 45 | | | 0.001 | 2 | | | 46 | | 0.006 | 2 | | | 58 | | 0.025 |
| 3 | 3 | | 0.02 | | 3 | | 7 | | | 0.02 | 3 | | 10 | | | 0.02 | 3 | | | 11 | | 0.04 | 3 | | | 12 | | 0.025 |
| 3 | 15 | | 0.01 | | 3 | | 16 | | | 0.03 | 3 | | 17 | | | 0.18 | 3 | | | 18 | | 0.05 | 3 | | | 20 | | 0.01 |
| 3 | 21 | | 0.001 | | 3 | | 23 | | | 0.02 | 3 | | 25 | | | 0.001 | 3 | | | 26 | | 0.006 | 3 | | | 27 | | 0.005 |
| 3 | 28 | | 0.001 | | 3 | | 29 | | | 0.035 | 3 | | 30 | | | 0.002 | 3 | | | 31 | | 0.002 | 3 | | | 32 | | 0.005 |
| 3 | 33 | | 0.01 | | 3 | | 34 | | | 0.05 | 3 | | 35 | | | 0.03 | 3 | | | 37 | | 0.011 | 3 | | | 39 | | 0.21 |
| 3 | 40 | | 0.25 | | 3 | | 41 | | | 0.005 | 3 | | 42 | | | 0.1 | 3 | | | 43 | | 0.3 | 3 | | | 44 | | 0.03 |
| 3 | 45 | | 0.001 | | 3 | | 58 | | | 0.95 | 4 | | 10 | | | 0.05 | 4 | | | 11 | | 0.4 | 4 | | | 12 | | 0.2 |
| 4 | 13 | | 0.118 | | 4 | | 14 | | | 0.009 | 4 | | 17 | | | 0.4 | 4 | | | 18 | | 0.15 | 4 | | | 22 | | 0.0015 |
| 4 | 23 | | 0.2 | | 4 | | 24 | | | 0.05 | 4 | | 25 | | | 0.05 | 4 | | | 26 | | 0.022 | 4 | | | 27 | | 0.005 |
| 4 | 30 | | 0.002 | | 4 | | 31 | | | 0.05 | 4 | | 32 | | | 0.04 | 4 | | | 33 | | 0.02 | 4 | | | 34 | | 0.03 |
| 4 | 35 | | 0.2 | | 4 | | 38 | | | 0.05 | 4 | | 39 | | | 0.2 | 4 | | | 40 | | 0.36 | 4 | | | 41 | | 0.15 |
| 4 | | 42 | | 0.38 | | 4 | | 43 | 0.12 | | | 4 | | 44 | 0.1 | | | 4 | 46 | | 0.05 | | | 4 | 51 | | 0.01 | |
| 4 | | 55 | | 0.02 | | 4 | | 56 | 0.01 | | | 4 | | 58 | 0.025 | | | 5 | 5 | | 0.2 | | | 5 | 6 | | 0.05 | |
| 5 | | 7 | | 0.03 | | 5 | | 8 | 0.25 | | | 5 | | 9 | 0.03 | | | 5 | 15 | | 0.1 | | | 5 | 19 | | 0.02 | |
| 6 | | 6 | | 0.05 | | 6 | | 7 | 0.5 | | | 6 | | 9 | 0.3 | | | 6 | 10 | | 0.1 | | | 6 | 14 | | 0.15 | |
| 6 | | 15 | | 0.45 | | 6 | | 16 | 0.32 | | | 6 | | 19 | 0.4 | | | 6 | 20 | | 0.05 | | | 6 | 21 | | 0.001 | |
| 6 | | 26 | | 0.39 | | 6 | | 29 | 0.002 | | | 6 | | 36 | 0.2 | | | 6 | 37 | | 0.818 | | | 6 | 40 | | 0.04 | |
| 6 | | 42 | | 0.08 | | 6 | | 50 | 0.01 | | | 6 | | 59 | 0.01 | | | 7 | 7 | | 0.35 | | | 7 | 9 | | 0.6 | |
| 7 | | 10 | | 0.2 | | 7 | | 14 | 0.38 | | | 7 | | 15 | 0.4 | | | 7 | 16 | | 0.52 | | | 7 | 19 | | 0.3 | |
| 7 | | 20 | | 0.6 | | 7 | | 21 | 0.1 | | | 7 | | 22 | 0.57 | | | 7 | 24 | | 0.2 | | | 7 | 25 | | 0.06 | |
| 7 | | 26 | | 0.44 | | 7 | | 27 | 0.78 | | | 7 | | 28 | 0.92 | | | 7 | 29 | | 0.3 | | | 7 | 30 | | 0.55 | |
| 7 | | 31 | | 0.1 | | 7 | | 32 | 0.35 | | | 7 | | 33 | 0.8 | | | 7 | 36 | | 0.5 | | | 7 | 37 | | 0.123 | |
| 7 | | 41 | | 0.005 | | 7 | | 42 | 0.1 | | | 7 | | 45 | 0.04 | | | 7 | 49 | | 0.03 | | | 7 | 50 | | 0.01 | |
| 7 | | 51 | | 0.01 | | 7 | | 59 | 0.5 | | | 8 | | 9 | 0.02 | | | 8 | 10 | | 0.03 | | | 8 | 14 | | 0.15 | |
| 8 | | 15 | | 0.025 | | 8 | | 16 | 0.04 | | | 8 | | 20 | 0.07 | | | 8 | 21 | | 0.2 | | | 8 | 22 | | 0.029 | |
| 8 | | 25 | | 0.01 | | 8 | | 26 | 0.004 | | | 8 | | 27 | 0.07 | | | 8 | 28 | | 0.008 | | | 8 | 29 | | 0.2 | |
| 8 | | 30 | | 0.025 | | 8 | | 31 | 0.1 | | | 8 | | 32 | 0.01 | | | 8 | 33 | | 0.05 | | | 8 | 34 | | 0.01 | |
| 8 | | 36 | | 0.25 | | 8 | | 37 | 0.029 | | | 8 | | 42 | 0.02 | | | 8 | 45 | | 0.001 | | | 8 | 47 | | 0.1 | |
| 8 | | 49 | | 0.015 | | 8 | | 51 | 0.01 | | | 8 | | 59 | 0.005 | | | 9 | 7 | | 0.05 | | | 9 | 9 | | 0.05 | |
| 9 | | 15 | | 0.01 | | 9 | | 16 | 0.045 | | | 9 | | 21 | 0.002 | | | 9 | 25 | | 0.04 | | | 9 | 26 | | 0.001 | |
| 9 | | 28 | | 0.001 | | 9 | | 29 | 0.32 | | | 9 | | 30 | 0.05 | | | 9 | 31 | | 0.08 | | | 9 | 34 | | 0.005 | |
| 9 | | 35 | | 0.01 | | 9 | | 36 | 0.05 | | | 9 | | 43 | 0.005 | | | 10 | 7 | | 0.02 | | | 10 | 16 | | 0.02 | |
| 10 | | 20 | | 0.01 | | 10 | | 21 | 0.002 | | | 10 | | 24 | 0.05 | | | 10 | 25 | | 0.05 | | | 10 | 29 | | 0.04 | |
| 10 | | 30 | | 0.01 | | 10 | | 31 | 0.02 | | | 10 | | 33 | 0.005 | | | 10 | 42 | | 0.01 | | | 10 | 49 | | 0.005 | |
| 11 | | 14 | | 0.005 | | 11 | | 17 | 0.01 | | | 11 | | 18 | 0.01 | | | 11 | 22 | | 0.02 | | | 11 | 23 | | 0.02 | |
| 11 | | 24 | | 0.001 | | 11 | | 25 | 0.015 | | | 11 | | 26 | 0.03 | | | 11 | 27 | | 0.03 | | | 11 | 28 | | 0.03 | |
| 11 | | 29 | | 0.001 | | 11 | | 30 | 0.02 | | | 11 | | 31 | 0.001 | | | 11 | 32 | | 0.12 | | | 11 | 33 | | 0.03 | |
| 11 | | 34 | | 0.05 | | 11 | | 35 | 0.01 | | | 11 | | 38 | 0.01 | | | 11 | 41 | | 0.02 | | | 11 | 42 | | 0.01 | |
| 11 | | 44 | | 0.01 | | 11 | | 45 | 0.04 | | | 11 | | 46 | 0.025 | | | 12 | 12 | | 0.2 | | | 12 | 14 | | 0.005 | |
| 12 | | 17 | | 0.04 | | 12 | | 18 | 0.04 | | | 12 | | 22 | 0.006 | | | 12 | 23 | | 0.2 | | | 12 | 25 | | 0.002 | |
| 12 | | 26 | | 0.075 | | 12 | | 31 | 0.042 | | | 12 | | 32 | 0.01 | | | 12 | 33 | | 0.01 | | | 12 | 34 | | 0.15 | |
| 12 | | 35 | | 0.25 | | 12 | | 38 | 0.01 | | | 12 | | 39 | 0.015 | | | 12 | 40 | | 0.005 | | | 12 | 41 | | 0.25 | |
| 12 | | 42 | | 0.06 | | 12 | | 43 | 0.02 | | | 12 | | 44 | 0.01 | | | 12 | 45 | | 0.002 | | | 12 | 46 | | 0.025 | |
| 13 | | 12 | | 0.005 | | 13 | | 17 | 0.005 | | | 13 | | 23 | 0.05 | | | 13 | 24 | | 0.02 | | | 13 | 25 | | 0.001 | |
| 13 | | 31 | | 0.001 | | 13 | | 38 | 0.05 | | | 13 | | 40 | 0.005 | | | 13 | 41 | | 0.005 | | | 13 | 42 | | 0.005 | |
| 13 | | 45 | | 0.002 | | 13 | | 46 | 0.025 | | | 13 | | 48 | 0.005 | | | 13 | 55 | | 0.025 | | | 14 | 17 | | 0.002 | |
| 14 | | 23 | | 0.025 | | 14 | | 25 | 0.002 | | | 14 | | 26 | 0.012 | | | 14 | 34 | | 0.2 | | | 14 | 35 | | 0.1 | |
| 14 | | 46 | | 0.001 | | 15 | | 12 | 0.001 | | | 15 | | 14 | 0.001 | | | 15 | 15 | | 0.005 | | | 15 | 16 | | 0.02 | |
| 15 | | 17 | | 0.001 | | 15 | | 18 | 0.3 | | | 15 | | 20 | 0.005 | | | 15 | 21 | | 0.01 | | | 15 | 22 | | 0.005 | |
| 15 | | 23 | | 0.01 | | 15 | | 24 | 0.005 | | | 15 | | 25 | 0.05 | | | 15 | 27 | | 0.03 | | | 15 | 29 | | 0.005 | |
| 15 | | 30 | | 0.025 | | 15 | | 31 | 0.01 | | | 15 | | 32 | 0.005 | | | 15 | 33 | | 0.015 | | | 15 | 34 | | 0.02 | |
| 15 | | 35 | | 0.05 | | 15 | | 38 | 0.15 | | | 15 | | 41 | 0.005 | | | 15 | 42 | | 0.005 | | | 15 | 47 | | 0.25 | |
| 15 | | 48 | | 0.012 | | 15 | | 49 | 0.1 | | | 15 | | 50 | 0.03 | | | 15 | 51 | | 0.17 | | | 15 | 52 | | 0.1 | |
| 15 | | 53 | | 0.21 | | 15 | | 54 | 0.7 | | | 15 | | 55 | 0.05 | | | 15 | 56 | | 0.2 | | | 15 | 57 | | 0.3 | |
| 15 | | 59 | | 0.035 | | 16 | | 12 | 0.1 | | | 16 | | 14 | 0.2 | | | 16 | 17 | | 0.01 | | | 16 | 20 | | 0.2 | |
| 16 | | 21 | | 0.612 | | 16 | | 22 | 0.32 | | | 16 | | 23 | 0.05 | | | 16 | 24 | | 0.2 | | | 16 | 25 | | 0.25 | |
| 16 | | 26 | | 0.004 | | 16 | | 27 | 0.01 | | | 16 | | 28 | 0.02 | | | 16 | 29 | | 0.015 | | | 16 | 30 | | 0.2 | |
| 16 | | 31 | | 0.5 | | 16 | | 32 | 0.25 | | | 16 | | 33 | 0.04 | | | 16 | 34 | | 0.02 | | | 16 | 37 | | 0.019 | |
| 16 | | 38 | | 0.105 | | 16 | | 41 | 0.1 | | | 16 | | 42 | 0.05 | | | 16 | 45 | | 0.3 | | | 16 | 46 | | 0.15 | |
| 16 | | 47 | | 0.3 | | 16 | | 48 | 0.35 | | | 16 | | 49 | 0.75 | | | 16 | 50 | | 0.817 | | | 16 | 51 | | 0.579 | |
| 16 | | 52 | | 0.16 | | 16 | | 53 | 0.36 | | | 16 | | 55 | 0.35 | | | 16 | 56 | | 0.259 | | | 16 | 57 | | 0.26 | |
| 16 | | 59 | | 0.34 | | 17 | | 14 | 0.1 | | | 17 | | 16 | 0.005 | | | 17 | 18 | | 0.05 | | | 17 | 20 | | 0.01 | |
| 17 | | 21 | | 0.002 | | 17 | | 22 | 0.016 | | | 17 | | 25 | 0.03 | | | 17 | 26 | | 0.004 | | | 17 | 27 | | 0.06 | |
| 17 | | 28 | | 0.02 | | 17 | | 29 | 0.035 | | | 17 | | 30 | 0.05 | | | 17 | 32 | | 0.1 | | | 17 | 33 | | 0.02 | |
| 17 | | 34 | | 0.02 | | 17 | | 35 | 0.1 | | | 17 | | 45 | 0.004 | | | 17 | 47 | | 0.2 | | | 17 | 48 | | 0.025 | |
| 17 | | 50 | | 0.01 | | 17 | | 51 | 0.02 | | | 17 | | 53 | 0.07 | | | 17 | 54 | | 0.05 | | | 17 | 56 | | 0.02 | |
| 17 | | 57 | | 0.024 | | 18 | | 12 | 0.01 | | | 18 | | 18 | 0.05 | | | 18 | 20 | | 0.004 | | | 18 | 21 | | 0.002 | |
| 18 | | 22 | | 0.002 | | 18 | | 23 | 0.065 | | | 18 | | 24 | 0.07 | | | 18 | 25 | | 0.08 | | | 18 | 26 | | 0.003 | |
| 18 | | 27 | | 0.01 | | 18 | | 30 | 0.01 | | | 18 | | 31 | 0.03 | | | 18 | 32 | | 0.05 | | | 18 | 34 | | 0.05 | |
| 18 | | 35 | | 0.05 | | 18 | | 38 | 0.25 | | | 18 | | 41 | 0.19 | | | 18 | 42 | | 0.01 | | | 18 | 46 | | 0.05 | |
| 18 | | 48 | | 0.025 | | 18 | | 50 | 0.002 | | | 18 | | 53 | 0.01 | | | 18 | 55 | | 0.05 | | | 18 | 56 | | 0.02 | |
| 18 | | 57 | | 0.011 | | 19 | | 25 | 0.005 | | | 19 | | 34 | 0.005 | | | 19 | 54 | | 0.039 | | | 20 | 21 | | 0.01 | |
| 20 | | 47 | | 0.05 | | 20 | | 48 | 0.1 | | | 20 | | 50 | 0.001 | | | 20 | 51 | | 0.001 | | | 20 | 52 | | 0.02 | |
| 20 | | 53 | | 0.05 | | 20 | | 56 | 0.025 | | | 20 | | 57 | 0.05 | | | 20 | 59 | | 0.1 | | | 21 | 47 | | 0.08 | |
| 21 | | 48 | | 0.06 | | 21 | | 52 | 0.02 | | | 21 | | 53 | 0.03 | | | 21 | 56 | | 0.022 | | | 21 | 57 | | 0.027 | |
| 21 | | 59 | | 0.01 | | 22 | | 20 | 0.001 | | | 22 | | 25 | 0.006 | | | 22 | 31 | | 0.02 | | | 22 | 46 | | 0.05 | |
| 22 | | 48 | | 0.05 | | 22 | | 50 | 0.01 | | | 22 | | 51 | 0.01 | | | 22 | 52 | | 0.1 | | | 22 | 53 | | 0.02 | |
| 22 | | 55 | | 0.1 | | 22 | | 56 | 0.05 | | | 22 | | 57 | 0.07 | | | 23 | 18 | | 0.05 | | | 23 | 21 | | 0.002 | |
| 23 | | 22 | | 0.025 | | 23 | | 23 | 0.05 | | | 23 | | 24 | 0.2 | | | 23 | 25 | | 0.136 | | | 23 | 29 | | 0.02 | |
| 23 | | 30 | | 0.01 | | 23 | | 34 | 0.15 | | | 23 | | 38 | 0.1 | | | 23 | 45 | | 0.5 | | | 23 | 46 | | 0.45 | |
| 23 | | 48 | | 0.25 | | 23 | | 53 | 0.15 | | | 23 | | 54 | 0.05 | | | 23 | 55 | | 0.1 | | | 23 | 56 | | 0.22 | |
| 23 | | 57 | | 0.148 | | 24 | | 25 | 0.002 | | | 24 | | 48 | 0.01 | | | 24 | 52 | | 0.04 | | | 24 | 53 | | 0.005 | |
| 24 | | 54 | | 0.025 | | 24 | | 56 | 0.02 | | | 25 | | 48 | 0.01 | | | 25 | 52 | | 0.04 | | | 25 | 53 | | 0.005 | |
| 25 | | 54 | | 0.025 | | 25 | | 56 | 0.02 | | | 26 | | 46 | 0.005 | | | 26 | 52 | | 0.05 | | | 26 | 53 | | 0.01 | |
| 26 | | 54 | | 0.025 | | 26 | | 56 | 0.01 | | | 26 | | 57 | 0.02 | | | 27 | 12 | | 0.001 | | | 27 | 17 | | 0.003 | |
| 27 | | 20 | | 0.005 | | 27 | | 21 | 0.025 | | | 27 | | 22 | 0.002 | | | 27 | 24 | | 0.01 | | | 27 | 25 | | 0.02 | |
| 27 | | 29 | | 0.02 | | 27 | | 30 | 0.03 | | | 27 | | 31 | 0.02 | | | 27 | 32 | | 0.015 | | | 27 | 34 | | 0.05 | |
| 27 | | 35 | | 0.015 | | 27 | | 38 | 0.024 | | | 27 | | 45 | 0.002 | | | 27 | 47 | | 0.01 | | | 27 | 49 | | 0.085 | |
| 27 | | 50 | | 0.094 | | 27 | | 51 | 0.082 | | | 27 | | 56 | 0.015 | | | 28 | 24 | | 0.0001 | | | 28 | 25 | | 0.0005 | |
| 28 | | 34 | | 0.001 | | 28 | | 45 | 0.0005 | | | 28 | | 46 | 0.002 | | | 28 | 48 | | 0.002 | | | 28 | 54 | | 0.02 | |
| 28 | | 56 | | 0.002 | | 28 | | 57 | 0.005 | | | 29 | | 24 | 0.0001 | | | 29 | 25 | | 0.0001 | | | 29 | 32 | | 0.0001 | |
| 29 | | 38 | | 0.001 | | 29 | | 45 | 0.0005 | | | 29 | | 46 | 0.0001 | | | 29 | 48 | | 0.002 | | | 29 | 53 | | 0.001 | |
| 29 | | 54 | | 0.001 | | 29 | | 55 | 0.002 | | | 29 | | 56 | 0.001 | | | 29 | 57 | | 0.005 | | | 30 | 24 | | 0.001 | |
| 30 | | 25 | | 0.0074 | | 30 | | 32 | 0.005 | | | 30 | | 38 | 0.008 | | | 30 | 45 | | 0.001 | | | 30 | 46 | | 0.004 | |
| 30 | | 47 | | 0.005 | | 30 | | 48 | 0.01 | | | 30 | | 52 | 0.003 | | | 30 | 53 | | 0.02 | | | 30 | 54 | | 0.01 | |
| 30 | | 55 | | 0.005 | | 30 | | 56 | 0.02 | | | 30 | | 57 | 0.03 | | | 31 | 24 | | 0.001 | | | 31 | 25 | | 0.008 | |
| 31 | | 32 | | 0.003 | | 31 | | 38 | 0.008 | | | 31 | | 45 | 0.002 | | | 31 | 46 | | 0.004 | | | 31 | 47 | | 0.005 | |
| 31 | | 48 | | 0.005 | | 31 | | 52 | 0.002 | | | 31 | | 53 | 0.01 | | | 31 | 54 | | 0.01 | | | 31 | 55 | | 0.01 | |
| 31 | | 56 | | 0.02 | | 31 | | 57 | 0.02 | | | 32 | | 32 | 0.001 | | | 32 | 38 | | 0.008 | | | 32 | 48 | | 0.002 | |
| 32 | | 52 | | 0.001 | | 32 | | 53 | 0.001 | | | 32 | | 55 | 0.01 | | | 32 | 56 | | 0.01 | | | 33 | 24 | | 0.0005 | |
| 33 | | 25 | | 0.005 | | 33 | | 32 | 0.001 | | | 33 | | 34 | 0.002 | | | 33 | 38 | | 0.008 | | | 33 | 45 | | 0.002 | |
| 33 | | 46 | | 0.005 | | 33 | | 48 | 0.005 | | | 33 | | 51 | 0.001 | | | 33 | 52 | | 0.001 | | | 33 | 53 | | 0.002 | |
| 33 | | 54 | | 0.01 | | 33 | | 55 | 0.005 | | | 33 | | 56 | 0.005 | | | 33 | 57 | | 0.015 | | | 34 | 24 | | 0.0005 | |
| 34 | | 25 | | 0.0005 | | 34 | | 34 | 0.002 | | | 34 | | 48 | 0.002 | | | 34 | 51 | | 0.001 | | | 34 | 54 | | 0.005 | |
| 34 | | 56 | | 0.002 | | 34 | | 57 | 0.005 | | | 35 | | 25 | 0.0002 | | | 35 | 54 | | 0.005 | | | 36 | 25 | | 0.009 | |
| 36 | | 34 | | 0.01 | | 37 | | 25 | 0.051 | | | 37 | | 34 | 0.05 | | | 38 | 12 | | 0.002 | | | 38 | 17 | | 0.002 | |
| 38 | | 20 | | 0.005 | | 38 | | 21 | 0.01 | | | 38 | | 23 | 0.02 | | | 38 | 24 | | 0.01 | | | 38 | 25 | | 0.025 | |
| 38 | | 29 | | 0.002 | | 38 | | 30 | 0.002 | | | 38 | | 31 | 0.004 | | | 38 | 32 | | 0.004 | | | 38 | 38 | | 0.004 | |
| 38 | | 45 | | 0.002 | | 38 | | 46 | 0.01 | | | 38 | | 48 | 0.005 | | | 38 | 49 | | 0.015 | | | 38 | 53 | | 0.01 | |
| 38 | | 55 | | 0.005 | | 38 | | 56 | 0.005 | | | 38 | | 57 | 0.01 | | | 39 | 24 | | 0.001 | | | 39 | 25 | | 0.001 | |
| 39 | | 48 | | 0.01 | | 39 | | 55 | 0.02 | | | 39 | | 56 | 0.01 | | | 40 | 11 | | 0.01 | | | 40 | 12 | | 0.01 | |
| 40 | | 17 | | 0.02 | | 40 | | 20 | 0.02 | | | 40 | | 21 | 0.01 | | | 40 | 23 | | 0.05 | | | 40 | 24 | | 0.02 | |
| 40 | | 25 | | 0.003 | | 40 | | 29 | 0.002 | | | 40 | | 30 | 0.002 | | | 40 | 32 | | 0.005 | | | 40 | 38 | | 0.024 | |
| 40 | | 41 | | 0.01 | | 40 | | 42 | 0.01 | | | 40 | | 45 | 0.01 | | | 40 | 46 | | 0.01 | | | 40 | 50 | | 0.008 | |
| 40 | | 51 | | 0.005 | | 41 | | 23 | 0.015 | | | 41 | | 24 | 0.01 | | | 41 | 25 | | 0.002 | | | 41 | 31 | | 0.001 | |
| 41 | | 32 | | 0.005 | | 41 | | 38 | 0.03 | | | 41 | | 41 | 0.02 | | | 41 | 45 | | 0.006 | | | 41 | 46 | | 0.013 | |
| 41 | | 48 | | 0.01 | | 41 | | 53 | 0.005 | | | 41 | | 55 | 0.078 | | | 41 | 56 | | 0.01 | | | 42 | 23 | | 0.005 | |
| 42 | | 24 | | 0.005 | | 42 | | 38 | 0.01 | | | 42 | | 45 | 0.002 | | | 42 | 46 | | 0.003 | | | 42 | 48 | | 0.002 | |
| 42 | | 55 | | 0.01 | | 42 | | 56 | 0.002 | | | 43 | | 12 | 0.02 | | | 43 | 17 | | 0.01 | | | 43 | 20 | | 0.01 | |
| 43 | | 21 | | 0.01 | | 43 | | 22 | 0.0035 | | | 43 | | 23 | 0.15 | | | 43 | 24 | | 0.123 | | | 43 | 25 | | 0.022 | |
| 43 | | 29 | | 0.002 | | 43 | | 30 | 0.012 | | | 43 | | 31 | 0.018 | | | 43 | 32 | | 0.0209 | | | 43 | 34 | | 0.011 | |
| 43 | | 35 | | 0.005 | | 43 | | 38 | 0.12 | | | 43 | | 41 | 0.21 | | | 43 | 43 | | 0.005 | | | 43 | 45 | | 0.061 | |
| 43 | | 46 | | 0.05 | | 43 | | 48 | 0.005 | | | 43 | | 50 | 0.008 | | | 43 | 51 | | 0.015 | | | 43 | 53 | | 0.025 | |
| 43 | | 55 | | 0.15 | | 43 | | 56 | 0.02 | | | 44 | | 12 | 0.001 | | | 44 | 21 | | 0.001 | | | 44 | 22 | | 0.0003 | |
| 44 | | 23 | | 0.025 | | 44 | | 24 | 0.01 | | | 44 | | 25 | 0.003 | | | 44 | 29 | | 0.001 | | | 44 | 31 | | 0.001 | |
| 44 | | 34 | | 0.002 | | 44 | | 38 | 0.02 | | | 44 | | 41 | 0.025 | | | 44 | 45 | | 0.01 | | | 44 | 46 | | 0.012 | |
| 44 | | 48 | | 0.01 | | 45 | | 23 | 0.02 | | | 45 | | 24 | 0.01 | | | 45 | 25 | | 0.001 | | | 45 | 34 | | 0.001 | |
| 45 | | 38 | | 0.01 | | 45 | | 41 | 0.005 | | | 45 | | 45 | 0.01 | | | 45 | 46 | | 0.02 | | | 45 | 48 | | 0.01 | |
| 45 | | 53 | | 0.001 | | 45 | | 56 | 0.002 | | | 46 | | 23 | 0.005 | | | 46 | 24 | | 0.002 | | | 46 | 34 | | 0.001 | |
| 46 | | 46 | | 0.01 | | 46 | | 48 | 0.008 | | | 46 | | 52 | 0.02 | | | 46 | 53 | | 0.001 | | | 46 | 55 | | 0.01 | |
| 47 | | 48 | | 0.01 | | 47 | | 52 | 0.025 | | | 47 | | 53 | 0.001 | | | 47 | 54 | | 0.025 | | | 48 | 48 | | 0.005 | |
| 48 | | 52 | | 0.005 | | 48 | | 53 | 0.001 | | | 49 | | 52 | 0.005 | | | 49 | 53 | | 0.002 | | | 51 | 51 | | 0.001 | |
| 54 | | 52 | | 0.14 | | 55 | | 52 | 0.018 | | | 56 | | 52 | 0.01 | | | 57 | 52 | | 0.05 | | | 58 | 52 | | 0.06 | |

^*^ “Pred” is the abbreviation of Predator
