# Supplementary material for: Biological interactions and cooperative management of multiple species
Source: PLoS One. 2017 Jun 29;12(6):e0180189. doi: 10.1371/journal.pone.0180189 (PMC5491148; doi:10.1371/journal.pone.0180189)
Supplement: S3 Table — (DOCX) [file pone.0180189.s004.docx]

**Table 3. Fleet landing in NCCME**

| Group name | bottom trawl (t/km^2^/year) | shrimp trawl (t/km^2^/year) | hake trawl (t/km^2^/year) | line, trap and pot (t/km^2^/year) | salmon fishery (t/km^2^/year) | crab pot (t/km^2^/year) | other (t/km^2^/year) |
| --- | --- | --- | --- | --- | --- | --- | --- |
| pandalid shp | 0 | 0.4174 | 0 | 0 | 0 | 0 | 0 |
| dungeness | 0 | 0 | 0 | 0 | 0 | 0.1927 | 0 |
| macrourids | 0.00243 | 0 | 0 | 0 | 0 | 0 | 0 |
| salmon | 0.00058 | 0 | 0.00168 | 0 | 0.105 | 0 | 0 |
| hake | 0 | 0 | 1.805 | 0 | 0 | 0 | 0 |
| skates | 0.0006 | 0 | 0 | 0 | 0 | 0 | 0 |
| dogfish | 0.00623 | 0 | 0 | 0.002 | 0 | 0 | 0 |
| sablefish | 0.0584 | 0.00062 | 4.95E-05 | 0.048 | 0 | 0 | 0 |
| POP | 0.018 | 2.00E-05 | 0 | 3.10E-06 | 0 | 0 | 0 |
| canary | 0.035437 | 0.001 | 0 | 0.000756 | 0 | 0 | 0.000497 |
| widow | 0.09818 | 0 | 0.0004 | 0 | 0 | 0 | 0 |
| yellowtail | 0.058561 | 0.005 | 0.00534 | 0.001335 | 0 | 0 | 0 |
| black | 0.0049 | 0 | 0 | 0.001 | 0 | 0 | 0.0005 |
| shelf rock | 0.0314 | 0.0058 | 0 | 0.014 | 0 | 0 | 0.0008 |
| slope rock | 0.027 | 0 | 0 | 0.0005 | 0 | 0 | 0.0064 |
| ssthorny | 0.042 | 0 | 0 | 0 | 0 | 0 | 0 |
| lsthorny | 0.043 | 0 | 0 | 0 | 0 | 0 | 0 |
| lingcod | 0.025 | 0.0002 | 0 | 0.0017 | 0 | 0 | 0.00067 |
| juv flat | 0 | 0 | 0 | 0 | 0 | 0 | 0 |
| english | 0.023 | 0 | 0 | 0 | 0 | 0 | 0 |
| petrale | 0.0202 | 0 | 0 | 0 | 0 | 0 | 0 |
| small flat | 0.0175 | 0 | 0 | 0 | 0 | 0 | 0 |
| rex | 0.0106 | 0 | 0 | 0 | 0 | 0 | 0 |
| dover | 0.076 | 0.0004 | 0 | 0 | 0 | 0 | 0 |
| arrowtooth | 0.055 | 0 | 0 | 0 | 0 | 0 | 0 |
| halibut | 0 | 0 | 0 | 0.00788 | 0 | 0 | 0 |
| albacore | 0 | 0 | 0 | 0 | 0.001 | 0 | 0 |
| coastal sharks | 0 | 0 | 0 | 0 | 0.001 | 0 | 0 |
