# Supplementary material for: Biological interactions and cooperative management of multiple species
Source: PLoS One. 2017 Jun 29;12(6):e0180189. doi: 10.1371/journal.pone.0180189 (PMC5491148; doi:10.1371/journal.pone.0180189)
Supplement: S4 Table — (DOCX) [file pone.0180189.s005.docx]

**Table 4. Fleet discarding in NCCME**

| Group name | | bottom trawl (t/km^2^) | shrimp trawl (t/km^2^) | hake trawl (t/km^2^) | line, trap and pot (t/km^2^) | salmon fishery (t/km^2^) | crab pot (t/km^2^) | other (t/km^2^) |
| --- | --- | --- | --- | --- | --- | --- | --- | --- |
| epibenthic | | 0.0095 | 0.00084 | 0 | 0 | 0 | 0 | 0 |
| large jellies | 0.0023 | | 0.0002 | 0 | 0 | 0 | 0 | 0 |
| dungeness | | 0.0035 | 0.000175 | 0 | 0 | 0 | 0 | 0 |
| tanner crb | | 0.0004 | 0 | 0 | 0 | 0 | 0 | 0 |
| cephalopods | | 0.0002 | 5.00E-05 | 0 | 0 | 0 | 0 | 0 |
| forage fish | | 0.0003 | 0.03517 | 0 | 0 | 0 | 0 | 0 |
| mesopelagics | | 0.0001 | 0 | 0 | 0 | 0 | 0 | 0 |
| benthic fish | | 0.0103 | 0.00735 | 0 | 0 | 0 | 0 | 0 |
| macrourids | | 0.0012 | 0 | 0 | 0 | 0 | 0 | 0 |
| mackerel | | 0.0002 | 0 | 0 | 0 | 0 | 0 | 0 |
| salmon | | 0.00058 | 0 | 0.00168 | 0 | 0 | 0 | 0 |
| hake | | 0.02 | 0.0734 | 1 | 0 | 0 | 0 | 0 |
| skates | | 0.0301 | 0.00156 | 0 | 0 | 0 | 0 | 0 |
| dogfish | | 0.036 | 0.00586 | 0 | 0 | 0 | 0 | 0 |
| sablefish | | 0.01168 | 0.00186 | 0 | 0 | 0 | 0 | 0 |
| POP | | 0.00334 | 3.80E-05 | 0 | 0 | 0 | 0 | 0 |
| canary | | 0.000436 | 0.0002 | 0 | 0 | 0 | 0 | 0 |
| widow | | 0.0187 | 0 | 0.0046 | 0 | 0 | 0 | 0 |
| yellowtail | | 0.01112 | 0.001 | 0 | 0 | 0 | 0 | 0 |
| black | | 0.0065 | 0 | 0 | 0 | 0 | 0 | 0 |
| shelf rock | | 0.0093 | 0.00227 | 0 | 0 | 0 | 0 | 0 |
| slope rock | | 0.0157 | 0.00558 | 0 | 0 | 0 | 0 | 0 |
| ssthorny | | 0.00167 | 0 | 0 | 0 | 0 | 0 | 0 |
| lsthorny | | 0.01147 | 0 | 0 | 0 | 0 | 0 | 0 |
| lingcod | | 0.0001 | 0.0002 | 0 | 0 | 0 | 0 | 0 |
| english | | 0.00311 | 0 | 0 | 0 | 0 | 0 | 0 |
| petrale | | 0.0046 | 0.000247 | 0 | 0 | 0 | 0 | 0 |
| small flat | | 0.0051 | 0.0137 | 0 | 0 | 0 | 0 | 0 |
| rex | | 0.007 | 0.00576 | 0 | 0 | 0 | 0 | 0 |
| dover | | 0.0114 | 0.0049 | 0 | 0 | 0 | 0 | 0 |
| arrowtooth | | 0.0103 | 0.00665 | 0 | 0 | 0 | 0 | 0 |
| halibut | | 0.002 | 0 | 0 | 0 | 0 | 0 | 0 |
| coastal sharks | | 0.0004 | 0 | 0 | 0 | 0 | 0 | 0 |
