# Supplementary material for: Biological interactions and cooperative management of multiple species
Source: PLoS One. 2017 Jun 29;12(6):e0180189. doi: 10.1371/journal.pone.0180189 (PMC5491148; doi:10.1371/journal.pone.0180189)
Supplement: S5 Table — (DOCX) [file pone.0180189.s006.docx]

**Table 5. Market price (US dollar)**

| Group name | bottom trawl (/unit biomass) | shrimp trawl (/unit biomass) | hake trawl (/unit biomass) | line, trap and pot (/unit biomass) | salmon fishery (/unit biomass) | crab pot (/unit biomass) | other (/unit biomass) |
| --- | --- | --- | --- | --- | --- | --- | --- |
| pandalid shp | | 7429.502 |  |  |  |  |  |
| dungeness | |  |  |  |  | 5291.04 |  |
| salmon | 4486.361 |  | 4486.361 |  | 4486.361 |  |  |
| hake |  |  | 220.46 |  |  |  |  |
| skates | 1263.98 |  |  |  |  |  |  |
| dogfish | 462.966 |  |  | 462.966 |  |  |  |
| sablefish | 5224.902 | 5224.902 | 5224.902 | 5224.902 |  |  |  |
| juv rock |  |  |  |  |  |  |  |
| POP | 3703.728 | 3703.728 |  | 3703.728 |  |  |  |
| canary | 2138.462 | 2138.462 |  | 2138.462 |  |  | 2138.462 |
| widow | 2138.462 |  | 2138.462 |  |  |  |  |
| yellowtail | 3350.992 | 3350.992 | 3350.992 | 3350.992 |  |  |  |
| black | 2138.462 |  |  | 2138.462 |  |  | 2138.462 |
| shelf rock | 2138.462 | 2138.462 |  | 2138.462 |  |  | 2138.462 |
| slope rock | 2138.462 |  |  | 2138.462 |  |  | 2138.462 |
| ssthorny | 2138.462 |  |  |  |  |  |  |
| lsthorny | 2138.462 |  |  |  |  |  |  |
| lingcod | 5776.052 | 5776.052 |  | 5776.052 |  |  | 5776.052 |
| english | 1477.082 |  |  |  |  |  |  |
| petrale | 4519.43 |  |  |  |  |  |  |
| small flat | 1219.879 |  |  |  |  |  |  |
| rex | 771.61 |  |  |  |  |  |  |
| dover | 837.748 | 837.748 |  |  |  |  |  |
| arrowtooth | 220.46 |  |  |  |  |  |  |
| halibut |  |  |  | 10339.57 |  |  |  |
| albacore |  |  |  |  | 2601.428 |  |  |
| coastal sharks | |  |  |  | 1179.461 |  |  |
